# Unified Stress–Strain Model of FRP-Confined Square and Circle Rubber Concrete Columns

**DOI:** 10.3390/ma15051832

**Published:** 2022-02-28

**Authors:** Yugui Cao, Guoxu Zhao, Yang Zhang, Can Hou, Ling Mao

**Affiliations:** 1 Hubei Key Laboratory of Roadway Bridge and Structure Engineering, Wuhan University of Technology, Wuhan 430070, China; caoyugui@163.com (Y.C.); zhaoguoxu@whut.edu.cn (G.Z.); yzhang229521@tongji.edu.cn (Y.Z.); 280219@whut.edu.cn (C.H.); 2 Sanya Science and Education Innovation Park, Wuhan University of Technology, Sanya 572024, China; 3 School of Civil Engineering and Architecture, Wuhan University of Technology, Wuhan 430070, China

**Keywords:** FRP, rubber concrete, stress–strain relationship, analytical model, confined concrete

## Abstract

Studying the stress–strain relationship of fiber-reinforced polymer (FRP)-confined rubber concrete (RuC) plays an important role in its application in engineering projects. Most of the existing stress–strain relationship models are established based on the test data of FRP-confined rubber concrete with circular cross-sections, and the effect of the section shape is not considered. Therefore, an analysis-oriented stress–strain model of FRP-confined circular and square rubber concrete columns was studied in this paper for the first time. A database that includes the rubber particle content and section shape on the peak stress-peak strain and axial–lateral strain relationship of FRP-confined rubber concrete was established by collecting 235 test data from the literature. By modifying the key parameters in the existing FRP-confined normal concrete stress–strain relationship model, a unified stress–strain relationship model of FRP-confined RuC with circular and square columns is established. The proposed model is verified, and a good accuracy of the model is proven.

## 1. Introduction

Rubber concrete (RuC) has the characteristic of low compressive strength compared to normal concrete, which limits its application in building structures. Although steel has been historically used to provide the required lateral confinement, fiber-reinforced polymers (FRP) have been used extensively over the last 20 years as a strengthening solution to enhance the ultimate compressive strain of concrete cylinders [1,2,3]. FRP has the advantages of high strength and good durability [4,5,6], and its lateral confinement effect can effectively improve the compressive strength of RuC [7], which makes it possible to apply RuC in engineering projects as structure building materials.

Thus, much experimental research and theoretical analyses on the mechanical properties of FRP-confined rubber concrete have been performed by many researchers. Moustafa et al. [8] conducted experimental research on the stress–strain relationship of FRP-confined rubber concrete and normal concrete under different strain rates. The results implied that FRP could provide greater confinement stress to rubberized concrete than normal concrete. Gholampour et al. [9] performed monotonic and cyclic axial compression tests of actively confined rubber concrete cylinders and found that the stress–strain relationship, peak stress, and peak strain of the specimens were significantly affected by a rubber volume replacement ratio and lateral confined stress. Based on the experimental data of FRP-confined rubber concrete with a circular section, Chan et al. [10] proved that the confined stress of FRP can improve the mechanical properties of RuC and that the compressive strength model of FRP-confined RuC was established. Bompa et al. [11] studied the mechanical behavior of FRP-confined rubber concrete with a circular cross-section and proposed a stress–strain model of FRP-confined rubber concrete columns. Cao et al. [12] studied the stress–strain relationship of FRP-confined rubber concrete cylinders under axial cyclic compression and proposed a stress–strain relationship model under axial cyclic compression. Compared with FRP-confined rubber concrete columns, there are few studies on FRP-confined rubber concrete square columns. Wang et al. [13] analyzed the mechanical properties of FRP-confined RuC square columns and proved that the cross-section shape has a significant effect on the confinement of FRP but did not establish the corresponding stress–strain relationship model. Although many scholars have proposed a stress–strain relationship model of rubber concrete, it has only been for FRP-confined rubber concrete circular columns, with no scholars having proposed a stress–strain model suitable for square-sectioned rubber concrete. This leads to a lack of corresponding theoretical basis and technical standards for the structure of FRP-confined RuC square columns, which limits further development and application. Therefore, exploring the axial compressive mechanical properties of the specimens of FRP-confined RuC square columns has a very important theoretical significance and engineering value, as rubber concrete can be applied to structures and provide a calculation basis for improving the seismic performance of structures.

This paper aims to propose a new model for predicting the stress–strain of FRP-confined circular and square rubber concrete columns. The database contained 235 experimental data and was built to establish the peak stress and peak strain model of FRP-confined columns. After evaluating the axial–lateral strain relationship model of FRP-confined RuC proposed by Chan et al. [10], the axial–lateral strain relationship model was established for FRP-confined circular and square rubber columns. Finally, the unified stress–strain model is proposed for FRP-confined circular and square rubber concrete columns, and the accuracy of the model is verified by the test data.

## 2. Database

**Figure 1:**
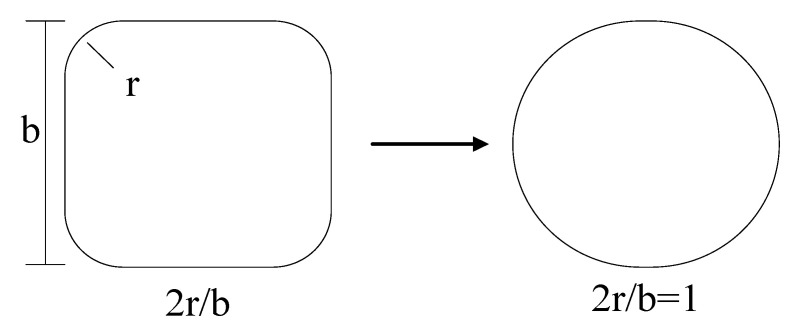
Transformation of uniform cross-section.

In this study, the database contains 235 stress–strain curves of FRP-confined circular and square rubber columns, including 75 square columns and 160 circular columns. There are two types of FRP-confined rubber concrete columns: concrete-filled FRP tubes (CFFT) and FRP-wrapped rubber concrete columns (“Wrap”). The test database mainly consists of three material types of FRP: carbon fiber-reinforced polymer (CFRP), glass fiber reinforced polymer (GFRP), and aramid fiber reinforced polymer (AFRP). Two specimen sizes are included: 100 × 200 mm and 150 × 300 mm. The rubber content (volume replacement ratio) *R_f_* varies from 0% to 75%. The strength, *f_co_*, and its corresponding axial strain, *ε_co_*, of unconfined rubber concrete are 6.8~69.5 MPa and 0.00069~0.0027, respectively. The section corner radius ratio (2*r*/*b*) of the specimens is between 0.2 and 1. If the section corner radius ratio is 2*r*/*b* = 1, then the specimen’s cross-section is circular, as shown in Figure 1. The database can be seen in Table 1.

## 3. Analytical Modeling

There are currently a large number of analytical stress–strain relationship models for FRP-confined concrete [22,23,24,25,26], which consist of three types of functions:

(1) The stress–strain relationship function of actively confined concrete with undetermined parameters (peak stress and peak strain) proposed by Richart [27] and Popovics [28], shown as Equations (1)–(3);

(2) The concrete type, the cross-section shape of the concrete columns, and the number of FRP layers can affect the lateral confinement of concrete, resulting in changes in the peak stress and peak strain. Therefore, it is necessary to define the functional expression of peak stress and peak strain, namely Equations (4) and (5);

(3) The relationship between the axial strain and lateral strain, as shown in Equation (6);

Based on the above three groups of formulas and the experimental stress–strain curve of FRP-confined rubber concrete, the stress–strain model of FRP-confined concrete can be obtained by using a nonlinear regression method or mathematical iterative method. In most existing models, the axial–lateral strain relationship *f*_a−l_ Equation (6) is generally obtained by nonlinear regression of the experimental data. The key parameters of the active confined model, i.e., peak stress and peak strain Equations (4) and (5), can be obtained by combining the axial–lateral strain model Equation (6) and the active confined stress–strain relationship model Equations (1)–(3).

The active confined concrete stress–strain model can be expressed as:

(1) $$f_{c}=\frac{f_{cc,w}xr}{r-1+x^{r}}$$

where

(2) $$x=\frac{\varepsilon_{c}}{\varepsilon_{cc,w}}$$

(3) $$r=\frac{E_{co}}{E_{co}-\frac{f_{cc,w}}{\varepsilon_{cc,w}}}$$

(4) $$f_{cc,w}\left(2r/b,f_{l}/f_{co}\right)=0$$

(5) $$\varepsilon_{cc,w}\left(2r/b,f_{l}/f_{co}\right)=0$$

The axial–lateral strain relationship can be written as:

(6) $$f_{a-l}\left(\varepsilon_{c},\varepsilon_{l},\sigma_{l}\right)=0$$

where $\varepsilon_{c},\varepsilon_{l},\sigma_{l},\sigma_{c}$ are the axial strain, lateral strain, confinement stress, and axial stress, respectively; $f_{cc,w}$ and $\varepsilon_{cc,w}$ are the peak stress and corresponding strain of concrete under active confinement, respectively; $E_{co}$ is the elastic modulus of concrete; and $f_{c}$ and $\varepsilon_{c}$ are the compressive stress and its corresponding strain, respectively. $E_{co}$ is the elastic modulus of rubber concrete when using Equations (1)–(3) to calculate the stress–strain relationship of FRP-confined rubber concrete, which can be calculated via Bompa’s model [29], which is suitable for rubber concrete:

(7) $$E_{c}=12,000{\left(f_{co}/10\right)}^{2/3}$$

### 3.1. Peak Stress and Peak Strain

In the analysis-oriented stress–strain model for FRP-confined concrete, the peak stress, $f_{cc,w}$, is usually modeled by using the ultimate strength *f_cc_* data of FRP-confined concrete specimens [22,30,31]. Therefore, the ultimate compressive strength *f_cc_* of FRP passively confined RuC is also used as the peak stress $f_{cc,w}$ of rubber concrete under active confinement. Most of the ultimate strength models of FRP-confined concrete are proposed based on Richart et al.’s model [27], where the function expression is:

(8) $$f_{cc}=f_{co}\left[1+k{\left(\frac{f_{l}}{f_{co}}\right)}^{\alpha }\right]$$

(9) $$f_{l}=\frac{2E_{frp}t_{frp}\varepsilon_{l}}{b}$$

where *f_cc_* and *f_co_* are the ultimate compressive strength of FRP-confined concrete and unconfined concrete, respectively, *k* and *a* are the coefficients to be determined, *f_l_* is the confinement stress of FRP, *E_frp_* is the elastic modulus of FRP material, *t_frp_* is the thickness of wrapped FRP, and *ε_l_* is the corner lateral strain of FRP material.

Existing studies show that the section shape of the specimens is one of the important factors affecting the compressive strength of FRP-confined normal concrete [32,33,34,35,36], FRP-confined rubber concrete [13,16], and FRP-confined recycled concrete [37,38]. The influence of the specimen section shape on FRP confinement can be modified by the parameter corner radius ratio (2*r*/*b*) [35,36], therefore Equation (4) can be rewritten as:

(10) $$f_{cc,w}=f_{\mathrm{co}}\left[1+a_{1}{\left(\frac{2r}{b}\right)}^{a_{2}}{\left(\frac{f_{l}}{f_{co}}\right)}^{f(R_{f})}\right]$$

where $f\left(R_{f}\right)$ is a coefficient related to the rubber content, which can be written as $f\left(R_{f}\right)=a_{3}+a_{4}R_{f}$, and *a*_1_, *a*_2_, *a*_3_, and *a*_4_ are the parameters that need to be determined. Through the nonlinear numerical regression analyses of Equation (10) based on the test data in Table 1, the values of *a*_1_, *a*_2_, *a*_3_, and *a*_4_ in Equation (10) can be obtained: *a*_1_ = 3.5, *a*_2_ = 0.3, *a*_3_ = 0.9, and *a*_4_ = −0.17. Therefore, Equation (10) becomes

(11) $$f_{cc,w}=f_{co}\left[1+3.5{\left(\frac{2r}{b}\right)}^{0.3}{\left(\frac{f_{l}}{f_{co}}\right)}^{\left(0.9-0.17R_{f}\right)}\right]$$

**Figure 2:**
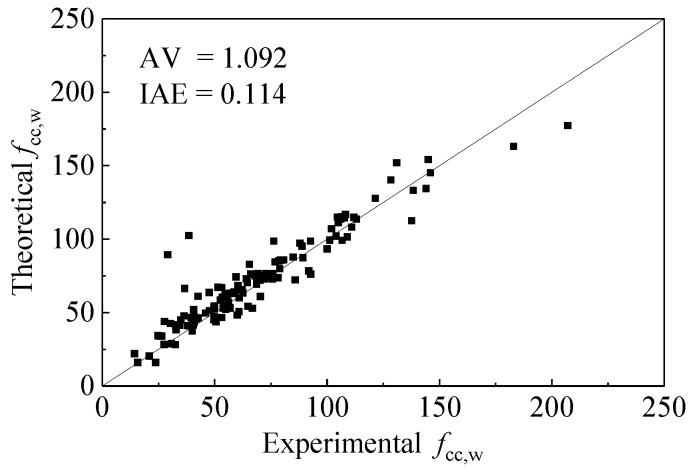
Performance of the peak stress model.

It can be seen that the proposed peak stress model of actively confined concrete has a good performance, as shown in Figure 2. AV and IAE are adopted in this work to estimate the accuracy of the peak stress model [12,32,39].

(12) AV=∑1nTheo iExpein

(13) $$\mathrm{IAE}=\frac{\sum_{1}^{n}{\left[{\left(Expe_{i}-Theo{\;}_{i}\right)}^{2}\right]}^{1/2}}{\sum_{1}^{n}\left|Expe_{i}\right|}$$

*Theo* and *Expe* are the theoretical value and experimental value, respectively, and *n* is the total number of data points; when the AV is closer to 1 and the IAE is closer to 0, the theoretical value is more accurate. Figure 2 shows that the proposed model has good performance in predicting the ultimate stress of rubber concrete circular and square columns. The AV and IAE are 0.117 and 1.055, respectively.

In the analysis-oriented stress–strain model, another governing parameter is the peak strain $\varepsilon_{cc,w}$ for actively confined concrete. The confinement stress, the strength of concrete, and the corresponding strain have an important influence on the peak stress of actively confined concrete [24,32]. The peak strain is usually linear with the peak stress [27], and the formula can be assumed to be:

(14) $$\varepsilon_{cc,w}=\varepsilon_{co}\left[1+b_{1}{\left(\frac{f_{l}}{f_{co}}\right)}^{b_{2}}\cdot {\left(\frac{2r}{b}\right)}^{b_{3}}\right]$$

where *b*_1_, *b*_1_, and *b*_3_ are the parameters that need to be determined. Equations (11) and (14) are substituted into Equation (1) through a nonlinear numerical regression analyses using the test database in Table 1. The coefficients *b*_1_, *b*_2_, and *b*_3_ are 18.7,1.09 and 0.44, respectively. Equation (14) then becomes:

(15) $$\varepsilon_{cc,w}=\varepsilon_{co}\left[1+18.7{\left(\frac{f_{l}}{f_{co}}\right)}^{1.09}\cdot {\left(\frac{2r}{b}\right)}^{0.44}\right]$$

**Figure 3:**
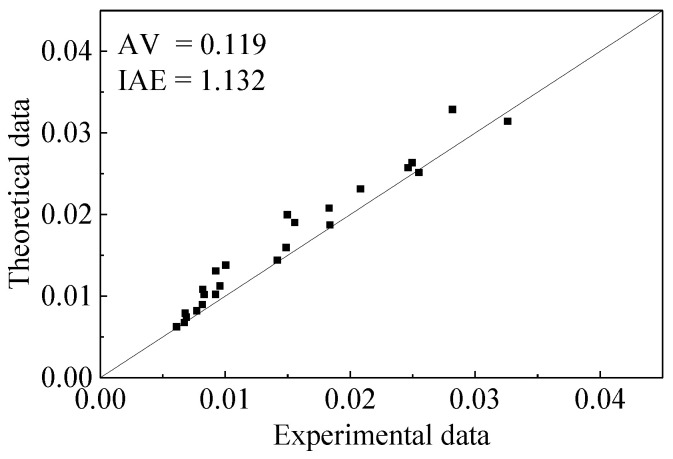
Performance of the peak strain model.

Because there are no peak strain data of square rubber concrete under active confinement, the accuracy of Equation (15) is evaluated by using the test data of circle rubber concrete under active confinement [9]. Figure 3 shows the evaluation results.

### 3.2. Axial–Lateral Strain Relationship

Chan et al. [10] modified Dai et al.’s [40] axial–lateral strain relationship of FRP-confined concrete by testing GFRP-wrapped rubber concrete and proposed an axial–lateral strain relationship of FRP-confined rubber concrete, as shown in the Equation (16). Cao et al. [12] proved that Chan et al.’s axial–lateral strain model [10] could be accurately used for circular rubber concrete under static loading.

(16) $$\frac{\varepsilon_{c}}{\varepsilon_{co}}=\left(1.0+8.0\frac{f_{l}}{f_{co}}\right)k_{R}\left[1.024{\left(\frac{\varepsilon_{l}}{\varepsilon_{co}}\right)}^{0.350}+0.089\left(\frac{\varepsilon_{l}}{\varepsilon_{co}}\right)\right]$$

where $\varepsilon_{c}$ is the axial compressive strain, $\varepsilon_{l}$ is the lateral strain, *f_l_* is the lateral confinement stress related to $\varepsilon_{l}$, and *k_R_* is the parameter related to rubber content, which is defined as:

(17) $$k_{R}=1-0.73R_{f}$$

**Figure 4:**
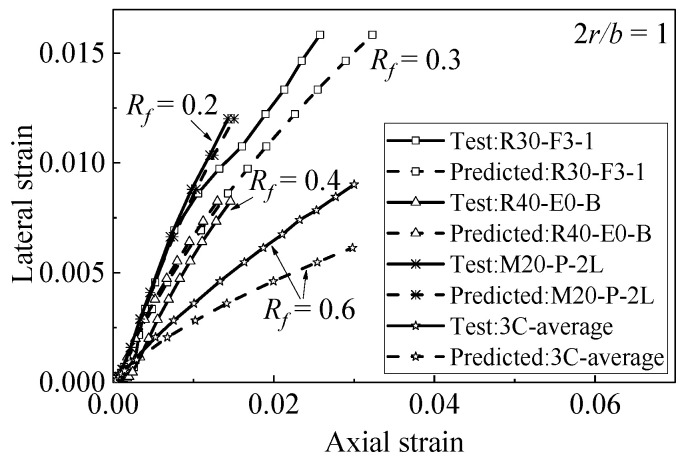
Performance of Chan et al.’s axial–lateral strain model [10].

Figure 4 shows the accuracy of Chan et al.’s [10] model in predicting the axial–lateral strain relationship of FRP-confined circle rubber concrete. It can be seen from Figure 4 that Chan et al.’s model has high accuracy in predicting the axial–lateral strain of circular rubber concrete columns (corner radius ratio is 1). However, Chan et al.’s model is obtained from the test data of FRP-confined rubber concrete with circular section, and the mechanical properties and deformation of FRP-confined square section concrete are different from those of circular concrete; therefore Chan et al.’s model is not suitable for FRP-confined square section rubber concrete and it is necessary to propose an axial–lateral strain model that is suitable for square-section rubber concrete.

In this work, the peak strain model of FRP-confined rubber concrete with circular and square cross-sections can be developed by modifying and extending the form of Equation (16), which is written as

(18) $$\frac{\varepsilon_{c}}{\varepsilon_{co}}=\left(1.0+8.0\frac{f_{l}}{f_{co}^{\prime }}\right)k_{R}\left[1.024{\left(\frac{\varepsilon_{l}}{\varepsilon_{co}}\right)}^{0.350}+0.089\left(\frac{\varepsilon_{l}}{\varepsilon_{co}}\right)\right]\varphi \left(\frac{2r}{b},R_{f}\right)$$

$\varphi \left(\frac{2r}{b},R_{f}\right)$ is a coefficient related to the corner radius and rubber content, which must be determined. $\varphi \left(\frac{2r}{b},R_{f}\right)={\left(\frac{2r}{b}\right)}^{f\left(R_{f}\right)}$, where $f\left(R_{f}\right)$ is a coefficient related to rubber content, which can be expressed as $f\left(R_{f}\right)=\alpha_{1}\cdot {\left(f_{l}/f_{co}\right)}^{\alpha_{2}}\cdot \left(1-\alpha_{3}R_{f}\right)$, where *a*_1_, *a*_2_, and d *a*_3_ are coefficients that need to be determined.

According to the data of the FRP-confined circular and square columns in Table 1, Equation (18) can be written in the form of Equation (19).

(19) εcεco=(1.0+8.0flfco′)kR[1.024(εlεco)0.350+0.089(εlεco)](2rb)0.187(flfco)0.364(1−6.143Rf)

**Figure 5:**
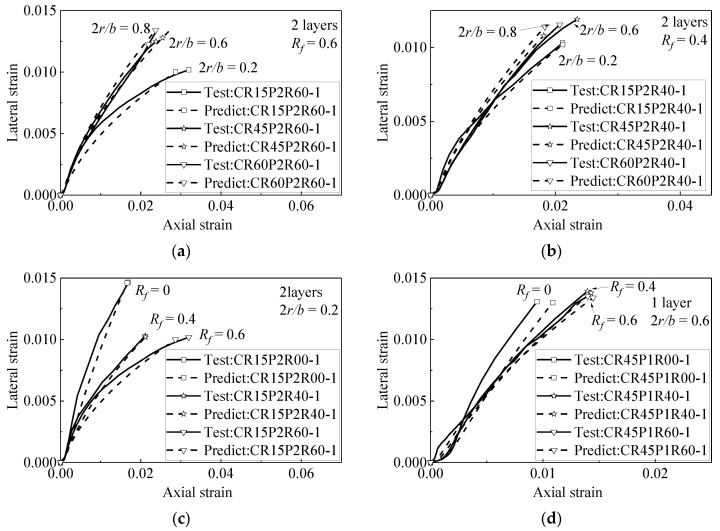
Performance of proposed axial–lateral strain model (data from reference [15]). (**a**) Specimens with different corner radii (**b**) Specimens with different corner radii (**c**) Specimens with different rubber content (**d**) Specimens with different rubber content.

Figure 5 is the evaluation result of Equation (19) and the evaluation data adopted from Zhang [15]. The data in Figure 5a,b show the change in the axial–lateral strain relationship with the corner radii under the same rubber content. Figure 5c,d compare the cases with the same corner radius. The higher the rubber content is, the smaller the hoop strain is. In other words, the proposed axial–lateral strain relationship model can be well-applied to square rubber concrete.

### 3.3. Stress–Strain Relationship

**Figure 6:**
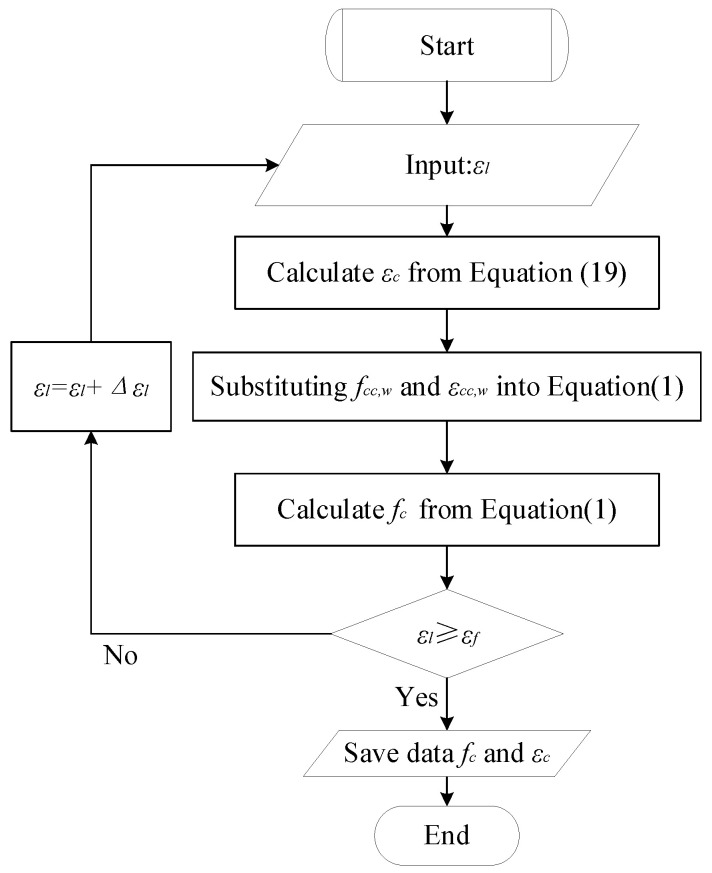
Flow chart of the procedure to generate the stress–strain model.

The proposed stress–strain model of FRP-confined concrete columns can be determined by an incremental iterative process. The iterative process can be expressed as follows and the programme flow chart is shown in Figure 6:

(1) Given a small initial lateral strain of concrete $\varepsilon_{l}$;

(2) The axial strain $\varepsilon_{c}$ corresponding to the lateral strain is calculated by Equation (19);

(3) The peak strain, $\varepsilon_{cc,w}$, and peak stress, $f_{cc,w}$, under confinement can be obtained;

(4) The peak stress, $f_{cc,w}$, and peak strain, $\varepsilon_{cc,w}$, are substituted into the active confined model Equation (1) to calculate *f_c_*;

(5) Increase the lateral strain $\varepsilon_{l}$ value and repeat steps (1)–(5) if the lateral strain is not greater than the FRP ultimate strain $\varepsilon_{f}$;

(6) The stress *f_c_* corresponding to each axial strain $\varepsilon_{c}$ can be obtained.

## 4. Model Prediction

**Figure 7:**
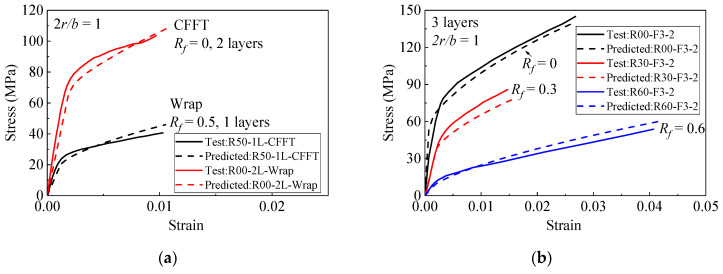

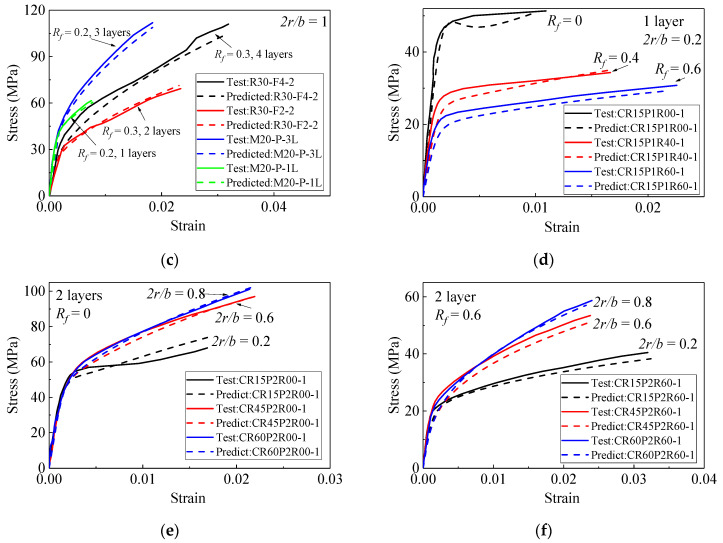
Performance of the proposed model for square normal concrete and RuC columns. (**a**) Specimens with different FRP retrofit methods. (**b**) Specimens with different rubber content. (**c**) Specimens with different FRP layers (**d**) Specimens with different FRP layers. (**e**) Specimens with different corner radii. (**f**) Specimens with different corner radii.

Based on the analysis, some selected experimental stress–strain curves collected from the literature in Table 1 are used to evaluate the performance of the different stress–strain relationships, as shown in Figure 7.

Figure 7 shows the stress–strain curves of FRP-confined rubber concrete and normal concrete with circular and square sections. Figure 7a–f shows the accuracy of the model in predicting FRP-confined concrete with different rubber contents, corner radii, and FRP layers. Figure 7a shows that the model has good accuracy in predicting different FRP wrapping methods, and Figure 7b shows that the theoretical value of the model has good agreement with the experimental value under different rubber content. It can be seen from Figure 7c that the prediction value of the model is very accurate in predicting the stress–strain relationship with different FRP layers. From Figure 7d, the accuracy of the FRP-confined square columns is still very accurate with the increase in rubber content under the small corner radius. Figure 7e,f show that the proposed analytical model has good accuracy in predicting the stress–strain model of plain concrete and rubber concrete with different corner radii. In general, the model proposed in this paper has high accuracy in predicting normal concrete square columns and rubber concrete square columns.

## 5. Conclusions

Based on the database of 235 FRP-confined rubber concrete circular and square columns, the axial–lateral strain relationship and stress–strain relationship of FRP-confined concrete with different rubber content and corner radii were analyzed. The following conclusions can be drawn:

1. There are no available axial–lateral strain relationships suitable for square rubber concrete. Based on the data of existing circular and square rubber concrete columns, an axial–lateral strain relationship model suitable for different corner radii and different rubber contents is established. The calculation results of this model were compared with the existing test data with a good performance.

2. An analytical model suitable for normal concrete and rubber concrete of circular and square cross-sections is established, and the model is verified by the test data, which shows that the analytical model has a good accuracy.

## Figures and Tables

**Table 1:** Database for model calibration.

Reference	Number of Specimens	FRP Type	*R_f_*	*ε_co_* (×10^−3^)	*f_co_* (MPa)	*b* × *h* (mm)	Retrofit Method	2*r*/*b*
Hou [14]	64	CFRP	0–0.6	1.6–2	16–45	150 × 300	Wrap	1
Zhang [15]	72	CFRP	0–0.6	1.1–2.2	10.3–37.2	150 × 300	Wrap	0.2–0.8
Chan et al. [10]	8	GFRP	0–0.75	2.2–2.7	13.9–53	150 × 300	Wrap	1
Cao et al [12]	12	CFRP	0–0.3	2.1–2.6	18.3–25.4	150 × 300	Wrap	1
Bompa [11]	22	AFRP	0–0.6	1.37–2.61	7.1–69.5	150 × 300	Wrap	1
Oprisan et al [16]	3	AFRP	0.4	0.69	10.97	100 × 200	Wrap	0.12
Hassanli et al. [17]	9	CFRP	0–0.4	1.7–1.95	20.7–32.0	100 × 200	Wrap	1
Youssf et al. [18]	12	CFRP	0–0.5	1.71–1.95	21.6–64.4	150 × 300	Wrap	1
Youssf et al. [19]	14	CFRP	0–0.2	1.67–2.43	39.2–62.5	100 × 200	CFFT	1
Tufail et al. [20]	9	CFRP	0.5	1.75	8–19	150 × 300	CFFT & Wrap	1
Raffoul et al. [21]	10	CFRP & AFRP	0.6	1.35	6.8–8.2	100 × 200 & 150 × 300	Wrap	1

Note: *R_f_* is the rubber content (volume replacement ratio), *f_co_* and *ε_co_* are the peak stress and its corresponding strain, respectively, *h* is the height of the specimen, *b* is the cross-section width, and *r* is the section corner radius.

## Data Availability

Data sharing is not applicable for this paper.
